# The thermal environment at fertilization mediates adaptive potential in the sea

**DOI:** 10.1002/evl3.215

**Published:** 2021-02-23

**Authors:** Evatt Chirgwin, Tim Connallon, Keyne Monro

**Affiliations:** ^1^ School of Biological Sciences Monash University Clayton Victoria Australia; ^2^ Cesar Australia Parkville Victoria Australia

**Keywords:** Additive genetic variation, environmental stress, evolution, external fertilization, gametes, global warming, marine invertebrates, phenotypic plasticity, reproduction, temperature

## Abstract

Additive genetic variation for fitness at vulnerable life stages governs the adaptive potential of populations facing stressful conditions under climate change, and can depend on current conditions as well as those experienced by past stages or generations. For sexual populations, fertilization is the key stage that links one generation to the next, yet the effects of fertilization environment on the adaptive potential at the vulnerable stages that then unfold during development are rarely considered, despite climatic stress posing risks for gamete function and fertility in many taxa and external fertilizers especially. Here, we develop a simple fitness landscape model exploring the effects of environmental stress at fertilization and development on the adaptive potential in early life. We then test our model with a quantitative genetic breeding design exposing family groups of a marine external fertilizer, the tubeworm *Galeolaria caespitosa*, to a factorial manipulation of current and projected temperatures at fertilization and development. We find that adaptive potential in early life is substantially reduced, to the point of being no longer detectable, by genotype‐specific carryover effects of fertilization under projected warming. We interpret these results in light of our fitness landscape model, and argue that the thermal environment at fertilization deserves more attention than it currently receives when forecasting the adaptive potential of populations confronting climate change.

**Impact Statement**

Natural populations need additive genetic variation for fitness at vulnerable life stages to adapt to climate change, and adaptive potential can depend on current environmental conditions as well as ones experienced by past stages or generations. Fertilization is the key life stage that links generations in most species, yet the impacts of fertilization environment on the adaptive potential at the vulnerable stages that then unfold during development are unknown and rarely considered. This knowledge gap is particularly concerning for the many aquatic species (including most fishes, amphibians, and marine invertebrates) that undergo fertilization and development in the external environment, directly exposed to rising water temperatures. We combine classic evolutionary theory with a novel breeding design to explore how external fertilization and development under projected ocean warming shape adaptive potential in early life for an intertidal ecosystem engineer, the marine tubeworm *Galeolaria caespitosa*. Our findings suggest that harmful carryover effects of fertilization under projected warming lower adaptive potential in early life, and failing to account for them may risk overestimating the adaptive potential of many species with similar biology. Our work has new implications not only for external fertilizers, but also for internally fertilizing ectotherms where fertilization is still vulnerable to ambient conditions, and argues that the thermal environment at fertilization deserves more attention than it currently receives when forecasting the adaptive potential of populations in a rapidly warming world.

Climate change is exposing populations to heightened stress and extinction risk (Scheffers et al. 2016). Populations that cannot move to escape, or cope in situ using phenotypic plasticity, can persist by undergoing evolutionary adaptation. Their potential to do so relies on genetic variation (specifically, additive genetic variation, although dominance and epistasis might sometimes contribute; Barton and Turelli 2004) for traits that enhance survival and reproduction (fitness) under the new conditions (Hoffmann and Sgrò 2011). Evidence that populations have the potential to adapt and persist under future scenarios of climatic stress is currently mixed (e.g., Kellermann et al. 2009; Kelly et al. 2013; Munday et al. 2017; Martins et al. 2019). However, tests of adaptive potential typically induce stress during the life stage in which the fitness component is expressed, which neglects potentially important carryover effects of stress experienced in past stages or generations (Sgrò and Hoffmann 1998; Chirgwin et al. 2018; Pujol et al. 2018). How such carryover effects shape adaptive potential—especially at life stages that are most vulnerable to stress and pose bottlenecks for population persistence—is largely unknown and in need of empirical testing.

There is a long tradition of using fitness landscape models to predict the extent to which a population genetically will vary in fitness and life history traits (Wright 1935; Tachida and Cockerham 1988; Price and Schluter 1991; Shaw and Shaw 2014). Simple Gaussian or quadratic fitness landscapes invoking selection to an optimum (Hansen 1997) predict that additive genetic variation for fitness—which determines the rate of adaptation (Fisher 1930)—should increase with the amount of genetic variation in traits affecting fitness, the distance between trait means and their optima, and the strength of stabilizing selection around the optima (see Box 1). Some or all of these factors are likely to change in altered environments (McGuigan and Sgrò 2009; Agrawal and Whitlock 2010; Martinossi‐Allibert et al. 2017; Fragata et al. 2019), leading to changes in adaptive potential. For example, environmental stress can increase adaptive potential through genotype‐environment interactions that increase genetic variation in quantitative traits affecting fitness (e.g., by releasing cryptic genetic variation; McGuigan and Sgrò 2009), or alter genetic correlations in ways that speed or limit adaptation when environmental conditions change (e.g., by making alleles more or less beneficial as stress progressively rises; Via and Lande 1985; Sgrò and Hoffmann 2004; Bell 2013). Stress can also alter the geometry of fitness landscapes (Box 1 and Fig. 1A; Martin and Lenormand 2006; Agrawal and Whitlock 2010), leading to changes in a population's distance to its optimum (i.e., the “lag load”; Fig. 1b‐C) and/or the strength of stabilizing selection (i.e., the standing genetic load; Fig. 1D‐E). These potential links between environmental change and fitness variation are implicit in classical fitness landscape theory, though their effects are rarely modeled explicitly (see: Martin and Lenormand 2006; Shaw and Shaw 2014).

**Figure 1:**
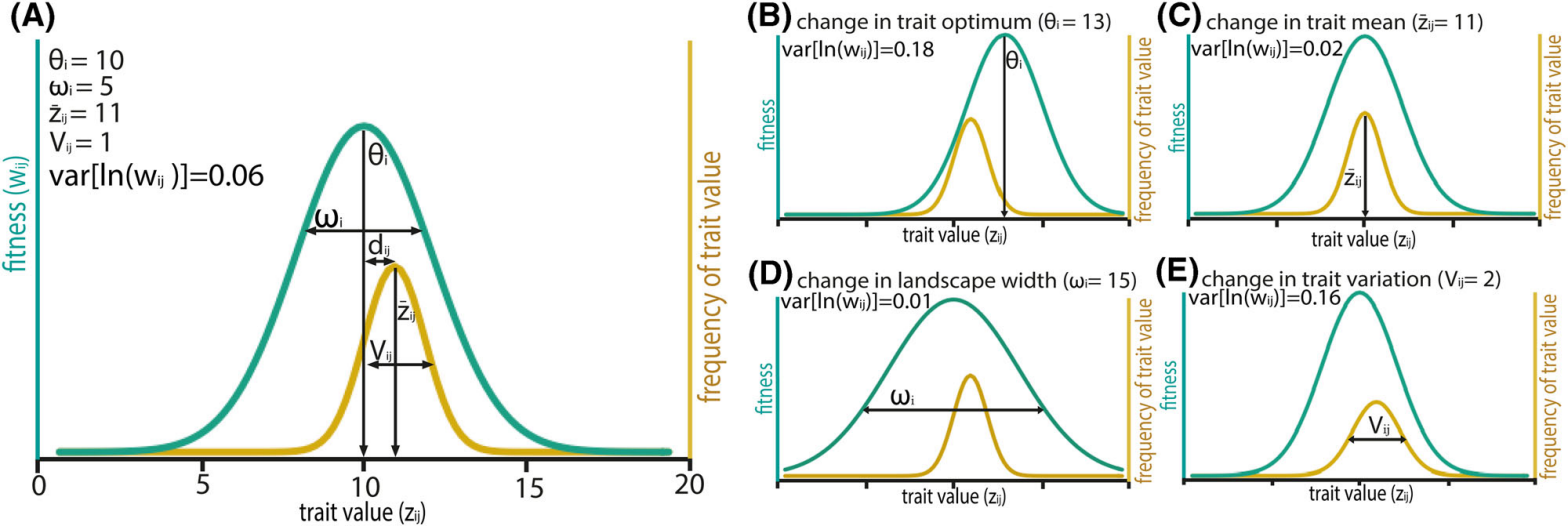
Fitness landscapes showing: (A) the key determinants ($d_{ij\;}^{2}\;,$ $V_{ij},$ $\omega_{i},$ $\theta_{i},$ ${\bar{z}}_{ij}$) of genetic variation for offspring fitness ($\mathrm{var}[\ln(w_{ij})]$; see Box 1), and (b‐e) four ways that environments at fertilization and development can affect such variation (results are based on equations from Box 1). Yellow curves show distributions of trait values, where *z_ij_* are values for offspring that develop in environment *i* and whose parents spawned in environment *j*. Green curves show landscapes (over all possible values of *z*) for offspring that develop in environment *i*.

For many organisms, adaptive potential is especially critical at early life stages (e.g., gametes, embryos, and larvae) whose vulnerability to stress makes them weak links in the life cycle (Zinn et al. 2010; Pandori and Sorte 2019). Such stages often sustain high mortality and experience different environmental conditions, allowing selection to modify allele frequencies and plasticity to modify allele expression as development unfolds (Bernasconi et al. 2004; Donohue 2014; Marshall et al. 2016; Postma and Ågren 2016). Fertilization, in particular, mechanistically links one generation to the next and is vital for the persistence of sexual populations, yet carryover effects of fertilization environment on the adaptive potential in early life are rarely considered, despite climatic stress posing major risks for gamete function and fertility in many taxa, and in external fertilizers most of all (Walsh et al. 2019). Unlike internal fertilizers, whose sperm and eggs interact entirely within the reproductive tract, many aquatic species (including most fishes, amphibians, and marine invertebrates) release sperm and eggs to fuse in the external medium where they are exposed directly to environmental stressors (Monro and Marshall 2015; Walsh et al. 2019; Chirgwin et al. 2020). Past work demonstrates that the environments experienced by gametes at fertilization can have carryover effects on offspring development and fitness (Parker et al. 2009; Ritchie and Marshall 2013; White et al. 2014), but the impacts on the adaptive potential in early life remain unexplored.

Here, we develop and test a simple fitness landscape model exploring the effects of environmental stress at fertilization and development on the adaptive potential in early life. We first extend the classic landscape framework to characterize how conditions at fertilization and development alter genetic variation and cross‐environment correlations for offspring fitness by altering adaptation, selection, and genotype‐environment interactions (Box 1). We then use our model to interpret the sensitivity of genetic variation for offspring survival to external fertilization and development under projected ocean warming (tested by factorial crosses of stage‐specific temperatures within a cross‐classified breeding design) in the marine tubeworm, *Galeolaria caespitosa*. We find that adaptive potential in early life is substantially reduced, to the point of being no longer detectable, by fertilization under projected warming, and explore possible reasons and implications in light of our model. Notwithstanding the limitations of quantitative genetic approaches like ours (e.g., Pujol et al. 2018), we present novel evidence that the thermal environment at fertilization may be a key, yet undervalued mediator of adaptive potential in vulnerable early life stages exposed to climatic stress.

**Box 1: A simple fitness landscape for environmental effects on the adaptive potential in early life**

We assume that genetic variation for offspring fitness (or a fitness component like survival) is affected by a single major trait whose expression depends on offspring genotype, developmental environment, and the environment of parents’ gametes during fertilization (see Supplementary Material for full model). For offspring that develop in the environment *i* and whose parents spawned in environment *j*, trait expression (*z_ij_*) is:

$$z_{ij}=x+y_{i}+b_{j}g+\varepsilon_{i}$$

where *x* is the environment‐independent genetic effect on the trait, *y_i_* is the genetic effect on the trait in the *i*th developmental environment, *b_j_g* is the genetic effect on the trait in the *j*th environment of parents’ gametes (*g* is a random variable and *b_j_* is a constant describing the magnitude and direction of the effect of the *j*th fertilization environment), and $\varepsilon_{i}$ is residual variation in environment *i*. We assume that $x,y_{i},g,and\;\;\varepsilon_{i}$ are independent, normally distributed random variables with means of $\bar{x},\;{\bar{y}}_{i},\bar{g},\;\;and\;{\bar{\varepsilon }}_{i}$ , and variances of $V_{x}\;\;,V_{yi\;\;},V_{g}\;,\;\mathrm{and}\;\;V_{\varepsilon i}$, respectively. Assuming that, $\bar{\varepsilon }\;=\;0$ the trait mean and genetic variance (respectively), are ${\bar{z}}_{\mathrm{ij}}\;\;=\bar{x}+{\bar{y}}_{i}+b_{j}\bar{g}\;\;\;\;\mathrm{and}\;\;\;\;\;V_{\mathrm{ij}}=V_{x}+V_{\mathrm{yi}}+b_{j}^{2}V_{g}$.

Offspring fitness in the environment *i* follows a Gaussian function with trait optimum $\theta_{i}$, and width $\omega_{i}$ (Fig. 1A). For convenience, we present results in a logarithmic scale, which approximate results in standard scale in populations that are reasonably well‐adapted to their environments (see Connallon and Matthews 2019). The mean fitness of offspring from environment *i* with parents from environment *j* is:

(1) $$E\;\left[\ln\left(w_{ij}\right)\right]=\ln\left(C_{i}\right)\;-\frac{d_{ij}^{2}}{2\omega_{i}}-\frac{V_{ij}+V_{\varepsilon i}}{2\omega_{i}}$$

where *C_i_* is the fitness of individuals expressing the optimal trait value, and $d_{ij}=\theta_{i}\;-\;{\bar{z}}_{ij}$ is the displacement of the trait mean from its optimum (see Wright 1935; Tachida and Cockerham 1988). Neglecting residual environmental variation, the genetic variance for fitness of offspring from environmental *ij* is:

(2) $$\mathrm{var}\;\left[\ln\left(w_{ij}\right)\right]=\frac{d_{ij}^{2}V_{ij}}{\omega_{i}^{2}}\;+\frac{V_{ij}^{2}}{2\omega_{i}^{2}}$$

Figure 1A visualizes how the five key quantities $\left(d_{\mathrm{ij}}^{2},V_{\mathrm{ij}},\omega_{i},\;\theta_{i},{\bar{z}}_{\mathrm{ij}}\right)$ of eq. (1) and (2) affect genetic variation for offspring fitness, while Figure 1B‐E visualizes four ways in which fertilization or developmental environments can affect fitness variation, including:

1. Changing the trait optimum $\left(\theta_{i}\right)$, which increases genetic variation for fitness if the optimum shifts away from the mean (Fig. 1B).
2. Changing the trait mean (${\bar{z}}_{\mathrm{ij}}$), which decreases genetic variation for fitness if the mean shifts toward the optimum (i.e., there is adaptive plasticity induced during development or carrying over from fertilization; Fig. 1C).
3. Changing the width around the optimum $\left(\omega_{i}\right)$, which decreases genetic variation for fitness if the width increases (i.e., stabilizing selection weakens; Fig. 1D).
4. Changing genetic variation for the trait (*V*_*ij*_), in which genetic variation for fitness increases with the trait's genetic variance (i.e., due to genotype‐environment interactions induced during development or carrying over from fertilization; Fig. 1E).

Extending the model to predict genetic correlations for fitness across fertilization or developmental environments, the genetic covariance for fitness across environments is:

(3) $$\begin{array}{c} \mathrm{cov}\;\left[\ln\left(w_{ij}\right),\ln\left(w_{kl}\right)\right]=\frac{\mathrm{cov}\left(z_{ij},z_{kl}\right)\left(2d_{ij}d_{kl}+\mathrm{cov}\left(z_{ij},z_{kl}\right)\right)}{2\omega_{i}\omega_{k}} \end{array}$$

where, for a given pair of populations, *i* and *k* are the developmental environments, *j* and *l* are the fertilization environments, $\mathrm{cov}(z_{ij},z_{kl})$ is the trait's genetic covariance across environments, and $\;d_{ij}=\;\theta_{i}-{\bar{z}}_{ij}\;and\;\;d_{kl}=\theta_{k}-{\bar{z}}_{kl}$ are the displacements of trait means from their optima. The cross‐environment genetic correlation for fitness is then:

(4) $$\rho_{ij,kl}=\frac{\mathrm{cov}\left[\ln\left(w_{ij}\right),\ln\left(w_{kl}\right)\right]}{\sqrt{\mathrm{var}\left[\ln\left(w_{ij}\right)\right]\mathrm{var}\left[\ln\left(w_{kl}\right)\right]}}$$

Eqs. ((3), (4)) imply that fitness correlations (covariances) are functions of trait correlations (covariances) and the degree to which each population is adapted to its environment. The correlation for fitness is strong and positive when the trait correlation is also strong and the direction of selection on the trait is the same across environments (i.e., $d_{ij}and\;d_{kl}$ have the same sign, so that $\;d_{ij}d_{kl}>0$). Changes in the trait's genetic basis (reducing $\mathrm{cov}(z_{ij},z_{kl})$) or direction of selection (so that $d_{ij}d_{kl}<0$) between environments lead to weakly positive or negative genetic correlations for fitness, implying genetic trade‐offs for fitness across environments.

## Methods

### STUDY SPECIES AND COLLECTION SITE

*Galeolaria caespitosa* (henceforth *Galeolaria*) is a calcareous tubeworm native to rocky shores of southeastern Australia, where it is an ecosystem engineer whose dense colonies of adult tubes provide habitat for endemic communities (Wright and Gribben 2017). Like many aquatic ectotherms, *Galeolaria* has sessile adults but planktonic gametes, embryos, and larvae, whose vulnerability to environmental stress makes them bottlenecks for population persistence under climate change (Pandori and Sorte 2019). Adult *Galeolaria* breed year‐round by spawning eggs and sperm into the external water column, where they must fuse for fertilization (Monro and Marshall 2016; Chirgwin et al. 2020), and where embryos and larvae also develop before eventually settling and recruiting into sessile adult populations. Survival to independence, the stage when larvae develop the capacity to swim and feed independently, is the most sensitive and reliable indicator of stress tolerance in *Galeolaria* and thus a key component of fitness in this species (Ross and Bidwell 2001; Chirgwin et al. 2015).

We sampled a population from the intertidal zone at Chelsea (Victoria, Australia) from April to June 2018, transferring individuals to Monash University in insulated aquaria. To reduce environmental differences among adults sampled at different times, we acclimatized adults at ∼16.5°C for 14–17 days (see Chirgwin et al. 2018) before extracting their gametes. To extract gametes, we induced spawning by removing adults from their tubes and placing them in Petri dishes with filtered seawater.

### FACTORIAL MANIPULATION OF ENVIRONMENTS AT FERTILIZATION AND DEVELOPMENT

**Figure 2:**
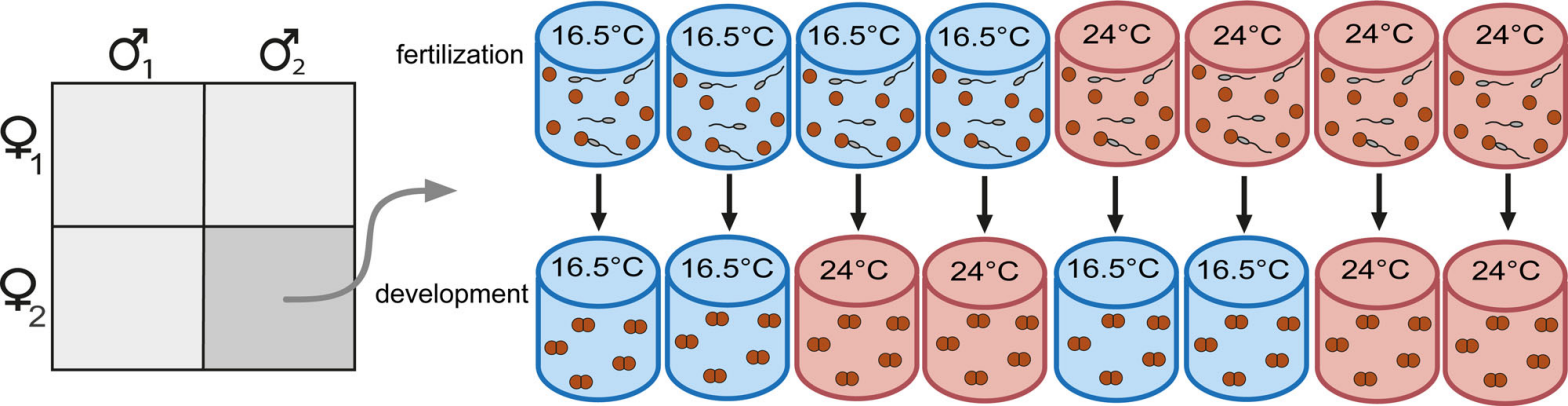
North Carolina II breeding design embedded in a factorial manipulation of life stage (fertilization and development to independence) and temperature (16.5°C and 24°C). Each sire‐dam cross was replicated in eight independent fertilization trials, four conducted at 16.5°C and four conducted at 24°C. Embryos from each trial developed at either the same temperature or the alternative temperature, so that each cross was replicated twice in each combination of fertilization and developmental environments.

Survival to independence was assayed in a factorial design, with environments at fertilization and development crossed at two temperatures representing current and projected warming at our study site. Here, sea‐surface temperature has ranged from 9 to 25°C over the past decade, averaging ∼16.5°C annually and ∼20.5°C in summer (CSIRO 2018). Mean sea temperature is projected to rise ∼2°C by 2050 and ∼3°C by 2070 (Hobday and Lough 2011; Mills et al. 2013). Given these projections, we conducted trials at 16.5°C (the current annual mean) and 24°C (currently rare in warmer months, but projected to become more common). To disentangle the effects of temperature at different life stages, our trials involved all four factorial combinations of fertilization and developmental temperatures (Fig. 2). Temperatures were maintained within 0.2°C of nominal values using drybath incubators.

### QUANTITATIVE GENETIC BREEDING DESIGN TO ESTIMATE GENETIC VARIATION FOR SURVIVAL

Within the factorial design above, we crossed gametes of males (sires) and females (dams) in a cross‐classified North Carolina II (NCII) breeding design (Fig. 2) to estimate additive genetic variation for survival at different temperatures. Such estimates are notoriously prone to imprecision and biasing by nonadditive genetic variation (dominance and epistasis) and shared environmental effects (e.g., of maternal environment; Kruuk and Hadfield 2007; Pujol et al. 2018). Though still subject to those caveats, our design leverages the scope for splitting egg‐clutches and ejaculates in *Galeolaria* to cross both sexes multiply and replicate crosses within and across environments, thereby improving precision and the partitioning of additive genetic variation from other genetic and shared environmental effects (Pederson 1972; Lynch and Walsh 1998). In each experimental block, we crossed sperm from two sires with eggs from two dams, yielding four families per block (Fig. 2). Each sire‐dam cross was replicated in eight independent fertilization, providing two replicates per cross for each of the four temperature treatments and 32 independent fertilization per block (Fig. 2). Our experiment had 28 blocks overall, yielding offspring from 56 sires, 56 dams, and 112 families.

### PROTOCOLS FOR FERTILIZATION AND DEVELOPMENT

Fertilization was initiated by adding ∼900 of a dam's eggs in 0.1 ml of filtered seawater to a vial holding ∼5 × 10^5^ of a sire's sperm in 1 ml of filtered seawater. For *Galeolaria*, these conditions maximize fertilization success while minimizing lethal polyspermy (Chirgwin et al. 2018). Before mixing, sperm and eggs were separately ramped to the desired temperature over 30 min. Sperm are activated by dilution, so were ramped at high concentration (10^7^ sperm/mL) to minimize aging (ramping period did not affect male fertility in pilot work; see Supplementary Material). Each fertilization trial ran for 30 minutes, maximizing fertilization success in pilot work. Each trial was agitated every 10 minutes to reduce oxygen depletion, then ended by thoroughly rinsing embryos through 25 μm Nitex mesh with filtered seawater.

Next, embryos were transferred from fertilization trials to developmental temperatures (Fig. 2). Because different fertilization temperatures led embryos to develop at different rates, we transferred them at the same developmental stage (2 to 8 cells, reached after ∼60 min at 24°C, and ∼90 min at 16.5°C). We did so to limit any confounding effects of implementing the development temperature at different stages, while still capturing ∼95% of embryonic development. For each replicate sire‐dam cross, we pipetted ∼30 embryos into a 1.5 ml vial of seawater, either maintained at the embryos’ fertilization temperature or ramped to the alternative temperature over ∼30 minutes (Fig. 2). Embryos are not oxygen‐limited while developing at this density (Chirgwin et al. 2018).

### SURVIVAL ASSAYS

All embryos developed at their nominal temperatures for 48 hours (they become independently swimming, feeding larvae after ∼24 hours). We used this period because previous work on *Galeolaria* suggests that 48 hours is the best time for reliably assessing survival to independence (Ross and Bidwell 2001). At the end of the developmental period, we added 0.1 ml of Lugol's solution to each vial to fix and stain the contents so larvae that survived to independence could be counted. Over 25,000 embryos (∼30 embryos × 2 replicates × 4 temperature treatments x 112 families) were counted overall.

### STATISTICAL ANALYSES

We used a multivariate linear mixed model, fitted via restricted maximum likelihood (REML) in ASReml‐R 3.0 (Butler et al. 2007), to estimate additive genetic, nonadditive genetic, and maternal environmental effects on offspring survival in different fertilization and developmental environments. In matrix form, we used:

$$y\;=\;X\beta \;+\;Z_{1}s+\;Z_{2}d\;+\;Z_{3}sd+Z_{4}b\;+\;\varepsilon$$

where *y* is survival, ***X*** is the design matrix for the fixed effects of fertilization and developmental environments (β), while **Z**_1_, **Z**_2_, **Z**_3_, and **Z**_4_ are design matrices for the random effects ***s***, ***d***, ***sd***, and ***b***, estimating sire variance (σ^2^ *_S_*), dam variance (σ^2^ *_D_*), sire × dam variance (σ^2^ *_SD_*), and block variance (σ^2^ *_B_*), respectively. The latter was modeled as a single variance, and each of the others as a block‐diagonal matrix with a separate submatrix per fertilization environment. Each submatrix modeled the variances for, and covariance between, survival in each developmental environment. Residual variance was modeled separately for each combination of fertilization and developmental environments. Residual diagnostics indicated no distributional problems, which was not the case for the equivalent animal model (hence our choice of the more classical approach here). We multiplied sire and sire × dam variances by four to calculate additive genetic and nonadditive genetic variances, respectively, and subtracted dam variances from sire ones to calculate maternal environmental variances (Fry, 2004). We tested each variance for significance by constraining it to 0 (or sire and dam variances to be equal, when testing maternal environmental variance) and using a likelihood ratio test to evaluate loss of fit relative to the original model.

To compare additive genetic, nonadditive genetic, and maternal environmental effects on survival between fertilization and developmental environments, we used likelihood ratio tests to compare the fits of nested models. In the case of fertilization, we tested if pooling each effect across fertilization environments resulted in worse fit than the original model with environment‐dependent effects. In the case of development, we first remodelled our data to estimate each effect as a block‐diagonal matrix with a separate submatrix per developmental temperature (instead of per fertilization temperature, as in our original model). This model estimated the same effects as before, but with a structure that allowed us to test if pooling each effect across developmental environments resulted in worse fit than the original model.

## Results

### EFFECTS OF FERTILIZATION AND DEVELOPMENTAL ENVIRONMENTS ON MEAN SURVIVAL

**Figure 3:**
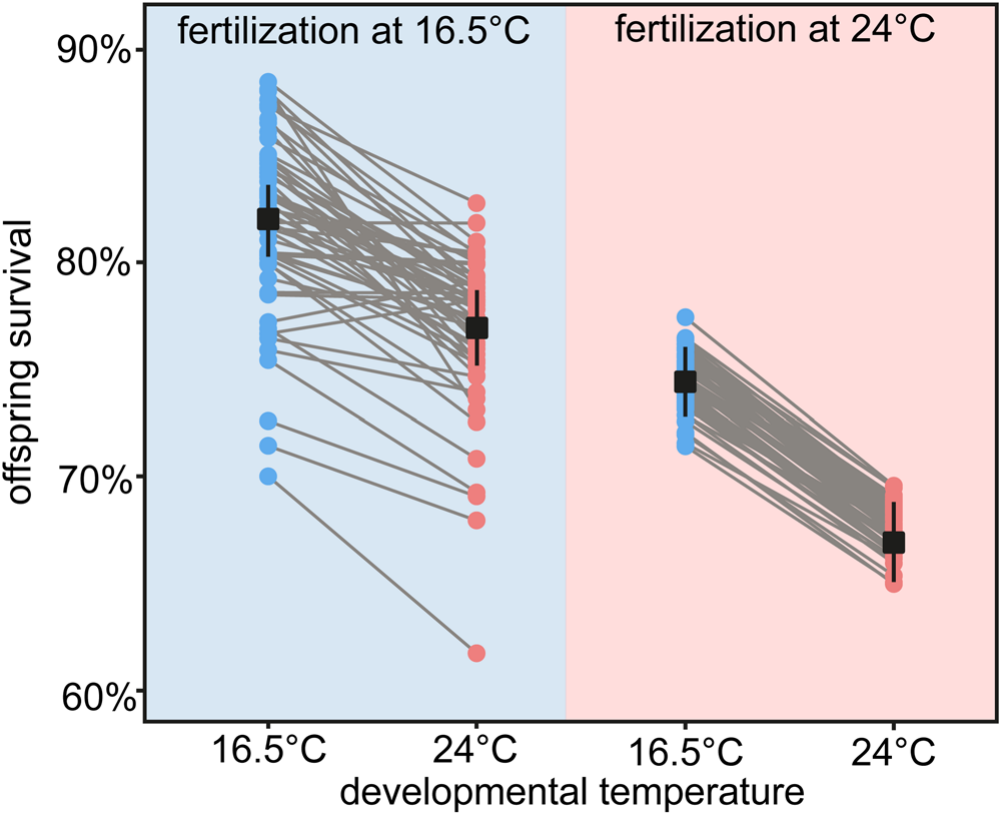
Effects of fertilization and developmental environments on offspring survival. Survival at 16.5°C is shown in blue and survival at 24°C is shown in pink. Black squares are overall means ± 1 standard error, and coloured dots are sire means. Variation among sire means at each temperature approximates additive genetic variation for survival at that temperature, and grey lines connecting sire means approximate the additive genetic covariance (or correlation) for survival across developmental temperatures.

Warmer fertilization and developmental temperatures significantly reduced mean offspring survival, without interacting in their effects (χ^2^ = 0.99, d.f. = 1, *P* = 0.32). Fertilization at warmer temperature reduced mean survival by ∼7% (Fig. 3; χ^2^ = 128.25, *df* = 1, *P* < 0.01), irrespective of developmental temperature, and development at warmer temperature reduced survival by ∼6% (Fig. 3; χ^2^ = 66.99, *df* = 1, *P* < 0.01), irrespective of fertilization temperature.

### EFFECTS OF FERTILIZATION AND DEVELOPMENTAL ENVIRONMENTS ON GENETIC VARIATION FOR SURVIVAL

Based on comparing the original model with environment‐dependent effects to models with effects pooled across environments, additive genetic variation for offspring survival was sensitive to fertilization temperature (χ^2^ = 9.02, *df* = 3, *P* = 0.03; Table 1a), but not developmental temperature (χ^2^ < 0.01, *df* = 3, *P* > 0.99; Table 1a). Offspring produced by fertilization at 16.5°C displayed significant amounts of additive genetic variation for survival at both developmental temperatures, and significantly positive additive genetic covariation across developmental temperatures (Table 1a; Fig. 3). For offspring produced by fertilization at 24°C, however, additive genetic variation for survival at either developmental temperature could not be distinguished from 0 (Table 1a; Fig. 3), nor could additive genetic covariation for survival across developmental temperatures.

**Table 1:** Genetic effects on offspring survival at current (16.5°C) and projected (24°C) fertilization and developmental temperatures. Effects of fertilization at 16.5°C are shown in plain text on the left, and effects of fertilization at 24°C are shown in italics on the right. Developmental temperatures are shown in rows and columns below each subheading. Estimates are ± 1 standard error; ^*^p<0.05 (see Table S1 for maternal environmental effects)

(a) Additive genetic variances and covariances					
Fertilization at 16.5°C			*Fertilization at 24°C*		
	16.5°C	24°C		*16.5°C*	*24°C*
16.5°C	0.014 ± 0.005^*^		*16.5°C*	*0.004 ± 0.004*	
24°C	0.008 ± 0.005^*^	0.013 ± 0.006^*^	*24°C*	*0.003 ± 0.003*	*0.003 ± 0.005*

(b) Nonadditive genetic variances and covariances					
Fertilization at 16.5°C			*Fertilization at 24°C*		
	16.5°C	24°C		*16.5°C*	*24°C*
16.5°C	0.010 ± 0.004^*^		*16.5°C*	*0.015* ± *0.005* ^*^	
24°C	0.011 ± 0.004^*^	0.017 ± 0.006^*^	*24°C*	*0.009* ± *0.005* ^*^	*0.018* ± *0.007* ^*^

Based on equivalent model comparisons, nonadditive genetic variation for offspring survival was also sensitive to fertilization temperature (χ^2^ = 23.22, *df*. = 3, *P* < 0.01; Table 1b), but not developmental temperature (χ^2^ < 0.01 d.f. = 3, *P* > 0.99; Table 1b). While nonadditive genetic variation for survival was detected in all treatments, it was generally higher for offspring produced by fertilization at 24°C than at 16.5°C (and when offspring developed at the warmer temperature, but not significantly so; Table 1b). Nonadditive genetic covariation for survival across developmental temperatures was consistently significant and positive, but marginally weaker when fertilization occurred at 24°C.

## Discussion

Populations need additive genetic variation for fitness at vulnerable life stages to adapt to climate change (Hoffmann and Sgrò 2011), and adaptive potential can depend on current environmental conditions as well as those experienced by past stages or generations (Munday et al. 2017). Yet the effects of fertilization environment on the adaptive potential at early life stages are rarely considered, despite their vulnerability to stress in many taxa, and despite fertilization mechanistically linking one generation to the next. Here in a marine external fertilizer—a group especially at risk of losing fertility due to rising temperatures (Walsh et al. 2019)—we show that fertilization under projected warming lowers adaptive potential in early life by negatively impacting additive genetic variation for survival and covariation for survival across current and projected temperatures, in addition to mean survival. Failing to account for such effects might therefore overestimate adaptive potential in species with similar biology (including most aquatic species; Blumer 1979; Monro and Marshall 2015), with implications for other studies (including our own) that conduct fertilization under benign conditions before assessing adaptive potential under stress. We interpret empirical results in light of fitness landscape models (Box 1), and argue that the fertilization environment deserves more attention than it currently receives when forecasting adaptive potential under climate change.

Understanding why warmer fertilization environment affects offspring survival in the ways detected here needs further work. Nonetheless, the impacts of fertilization environment on genetic variation for survival are inconsistent with temperature‐dependent shifts in the trait optimum (Fig. 1B) or width of the fitness landscape (Fig. 1D), since both phenomena depend on the developmental environment of offspring (Box 1). Such impacts are also inconsistent with temperature‐dependent shifts in trait means arising from adaptive plasticity (Fig. 1C). For example, while adaptive plasticity carrying over from fertilization (and decreasing | *d_ij_*|) will tend to reduce genetic variance for fitness, it will also increase mean survival, which is opposite to what was observed. The likely reason, in light of our model, is that genotype‐environment interactions carrying over from fertilization at warmer temperature decrease additive genetic variation (and covariation) for survival in warmer conditions (i.e., opposite to the change to V_ij_ shown in Fig. 1E), and to an extent that offsets the anticipated increase due to lower survival in those conditions. Carryover effects of fertilization environment might therefore be maladaptive but genotype‐specific, and might variously signal heat‐induced DNA damage, epigenetic effects, or selection in gametes after spawning (Bernasconi et al. 2004; Lewis and Aitken 2005; Immler and Otto 2018; Lymbery et al. 2020). Such biological mechanisms await future testing.

Our results add to mounting evidence that future climate change may enhance nonadditive genetic effects on fitness and related traits (Lymbery and Evans 2013; Chirgwin et al. 2017, 2018; Rudin‐Bitterli et al. 2018). Nonadditive genetic variation for offspring survival was evident in all environments but increased with warmer environment at fertilization, suggesting that dominance and/or epistasis influence offspring survival and may do so more under projected warming. The broader implications for adaptation remain unclear (Hansen 2015; Hill 2017) but, at a minimum, stronger nonadditive genetic effects on fitness at vulnerable life stages may make demographic and evolutionary dynamics less predictable if more of the variation for fitness depends on allele combinations that are shuffled by random segregation and recombination from one generation to the next (Falconer and Mackay 1996; Puurtinen et al. 2005). In theory, nonadditive genetic variation may contribute to adaptation if converted to additive genetic variation by drift after population bottlenecks (Goodnight 1988; Barton and Turelli 2004), but much of the converted variation is expected to be deleterious and quickly removed by selection unless it helps populations to new, evolutionarily‐stable states (e.g., persistence rather than extinction; Barton and Turelli 2004). Currently, however, evidence that it does so remains equivocal (van Heerwaarden et al. 2008).

The net evolutionary impacts of environmental stress at different life stages remain poorly understood (Beaman et al. 2016; Marshall et al. 2016). Here in *Galeolaria*, fertilization and development under projected warming impose similar costs on offspring survival, yet the former environmental context has a greater effect on genetic variation for survival than the latter context. Thus, in *Galeolaria* at least, adaptive potential in early life is more sensitive to the life stage that stress occurs than to the amount of stress itself, with implications for interpreting such potential when stress is imposed after benign conditions earlier in the life cycle (e.g., Chirgwin et al. 2015). Adaptive potential in early life may also be sensitive to the parental environment (Munday et al. 2017), which could not be considered here. However, previous work on *Galeolaria* suggests that parental exposure to warming actually improves the mean survival of offspring, while weakly reducing additive genetic variation for survival (Chirgwin et al. 2018). Hence, parental effects might buffer offspring against the kind of decline in mean survival under warming seen here, but seem unlikely to compensate for the added loss of adaptive potential.

Our results also add to evidence that gametes exposed to environmental stress produce fewer or poorer offspring (Parker et al. 2009; Byrne 2011, but see Ritchie and Marshall 2013), whereas stress at diploid life stages more often induces plasticity that buffers later stages or generations against stress (Sgrò et al. 2016; Kellermann et al. 2017). There are various reasons why gametes might be more sensitive than diploid stages to stress, including smaller size (Klockmann et al. 2017), lower ploidy (Scholes and Paige 2015), and reduced repertoire of stress‐response mechanisms (e.g., epigenetic changes, expression of heat‐shock proteins; Feder and Hofmann 1999; Donkin and Barrès 2018). The latter, in particular, can mask genetic variation during development (Queitsch et al. 2002), and similar masking might explain why genetic variation for survival was less sensitive to developmental temperature than fertilization temperature here. Nonetheless, caution is needed in interpreting quantitative changes in genetic variation across environments, given the imprecision of quantitative genetic estimates (Kruuk and Hadfield 2007; Pujol et al. 2018), and the risk that controlled laboratory conditions only approximate ecologically‐relevant contributions to early survival in nature. Such limitations of our approach (which remains one of the few options for species like *Galeolaria* that are not yet tractable to pedigree analysis in wild populations; Pemberton 2008) by no means invalidate our inferences about the environment‐dependence of genetic variation in early life, but may warrant treating them as more qualitative than quantitative (Pujol et al. 2018).

Together, our findings suggest that the thermal environment at fertilization warrants more attention in a rapidly warming world. In *Galeolaria*, harmful carryover effects of fertilization under projected ocean warming not only reduce a key component of fitness in early life, but also the potential to recover fitness through evolutionary adaptation to warming. This is in marked contrast to the usual expectation that stress increases adaptive potential (Rowiński and Rogell 2017). Whilst external fertilizers like *Galeolaria* are considered most vulnerable to the impacts of future warming on fertility, our work also has implications for internally fertilizing ectotherms in which fertilization is still vulnerable to ambient conditions (Walsh et al. 2019). Since fertilization is vital for the persistence of all sexual populations, overlooking the evolutionary impacts of fertilization environment may lead us to misjudge the vulnerability and adaptive potential of populations confronting climate change.

## AUTHOR CONTRIBUTIONS

E.C. and K.M. conceived and designed the study. T.C. derived the models. E.C. collected the data. E.C. and K.M. performed analyses and wrote the first draft of the manuscript. All authors contributed to revisions.

## DATA ARCHIVING

Data and R‐script have been deposited in Dryad (https://doi.org/10.5061/dryad.wpzgmsbm9).

Associate Editor: A. Charmantier

## Supporting information


**Table S1**. Maternal environmental effects on offspring survival at current (16.5°C) and projected (24°C) fertilisation and developmental temperatures.
**Figure S1**. No impact of sperm ramping period on mean fertilisation success (±S.E.).

## Data Availability

Data and code have been deposited on Dryad (https://doi.org/10.5061/dryad.wpzgmsbm9).
