# Supplementary material for: The thermal environment at fertilization mediates adaptive potential in the sea
Source: Evol Lett. 2021 Feb 23;5(2):154–63. doi: 10.1002/evl3.215 (PMC8045945; doi:10.1002/evl3.215)
Supplement: Supplementary file 1 — Table S1. Maternal environmental effects on offspring survival at current (16.5°C) and projected (24°C) fertilisation and developmental temperatures. Figure S1. No impact of sperm ramping period on mean fertilisation success (±S.E.). [file EVL3-5-154-s001.docx]

**Model appendix and supplementary material**

**Model appendix**

**General assumptions of the model**

We assume, for simplicity, that variation in survival is affected by the expression of a single major trait whose expression depends on both the genotype of the offspring and the fertilization environment of the parents’ gametes. Additive genetic variance can be partitioned into three independent components of trait variation:

1. Genetic variation with phenotypic effects that are independent of the environmental conditions of parents or of offspring (hereafter, “environment-independent” genetic variation).
2. Genetic variation with phenotypic effects that depend on the developmental environment of offspring (hereafter, the “cryptic” genetic variation).
3. Genetic variation with phenotypic effects that depend on the environment of fertilization (hereafter, “fertilization-dependent” genetic variation). Effects of both natural selection among gametes and non-genetic parental effects are subsumed into this variance component.

Since new mutations in gametes do not contribute to covariances between siblings, mutational effects will be subsumed into the residual environmental variation and we can therefore ignore effects on estimates of *V_A_*. Although genetic drift may also contribute to allele frequency changes that can affect *V_A_*, prior theory predicts that such effects are likely to be small in outbred populations (see Barton and Turelli 2004), and we therefore ignore it.

**Genetic and environmental basis of trait expression**

Consider individuals that develop in environment *i*, and whose parents spawned in environment *j*. An individual’s trait expression (*z_ij_*) is a function of the three components of additive genetic variance plus residual variance according to the following linear model:

$$z_{ij}=x+y_{i}+b_{j}g+\varepsilon_{i}$$

The elements of the linear model include: (1) *x* as the effect of environment-independent genetic variation on the individual’s trait expression, (2) *y_i_* as the effect of cryptic genetic variation in the *i*th developmental environment, which we assume is uncorrelated between developmental environments (*e.g*., cov(*y*_1_, *y*_2_) = 0 between developmental environments 1 and 2), (3) *b_j_g* as the fertilization-dependent genetic effect, in which *g* is random variable reflecting genetic variation that is sensitive the fertilization environment and *b_j_* is a constant representing the effect of the *j*th fertilization environment on the expression of *g*, and (4) *ε_i_* is the residual variation in the *i*th developmental environment. We assume that *x*, *y_i_*, and *g* are independent, normally distributed random variables with means of $\bar{x}$, $\bar{y}_{i}$, and $\bar{g}$ and variances of $V_{x}$, $V_{yi}$, and $V_{g}$ (respectively); *ε_i_* is normally distributed with mean of $\bar{\varepsilon}_{i}=0$ and variance of $V_{\varepsilon i}$. Consequently, the trait mean and additive genetic variance in offspring fertilized in environment *j* and developing in environment *i* are:

$$\bar{z}_{ij}=\bar{x}+\bar{y}_{i}+b_{j}\bar{g}$$

$$V_{ij}=V_{x}+V_{yi}+b_{j}^{2}V_{g}$$

**Effect of trait expression on a fitness component**

The fitness component of offspring that are reared in environment *i* (for simplicity, the fitness component is hereafter referred to as “fitness”) is a Gaussian function of trait expression:

$$w_{i}=C_{i}\exp\left( -\frac{\left( \theta_{i}-z_{ij} \right)^{2}}{2\omega_{i}} \right)$$

where *θ_i_* is the optimal trait value and *ω_i_* is the width of the fitness surface in the *i*th developmental environment; *C_i_* is the mean survival of individuals that express the optimal trait value.

For convenience, we work in log scale in the following results, and note that the following variances and covariances correspond approximately to results in standard scale provided populations are reasonably well adapted to their environments (see Connallon and Matthews 2019).

**Mean fitness**

The mean population fitness in environment *ij* is:

$$E\left[ \ln\left( w_{ij} \right) \right]=\ln\left( C_{i} \right)-E\left[ \frac{\left( \theta_{i}-z_{ij} \right)^{2}}{2\omega_{i}} \right]=\ln\left( C_{i} \right)-E\left[ \frac{d_{ij}^{2}}{2\omega_{i}}-\frac{d_{ij}\left( z_{ij}-\bar{z}_{ij} \right)}{\omega_{i}}+\frac{\left( z_{ij}-\bar{z}_{ij} \right)^{2}}{2\omega_{i}} \right]=\ln\left( C_{i} \right)-\frac{d_{ij}^{2}}{2\omega_{i}}-\frac{V_{ij}+V_{\varepsilon i}}{2\omega_{i}}$$

where $d_{ij}=\theta_{i}-\bar{z}_{ij}$ is the average displacement of the trait from the optimum (see Wright 1935; Tachida and Cockerham 1988).

**Genetic variance for fitness**

Neglecting residual environmental variation, the genetic variance for fitness of individuals from environmental combination *ij* is:

$$\mathrm{var}\left[ \ln\left( w_{ij} \right) \right]=\frac{V_{ij}\left( 2d_{ij}^{2}+V_{ij} \right)}{2\omega_{i}^{2}}=\frac{d_{ij}^{2}V_{ij}}{\omega_{i}^{2}}+\frac{V_{ij}^{2}}{2\omega_{i}^{2}}$$

(*e.g.*, Connallon and Matthews 2019). The final expression partitions the genetic variance for fitness into components that depend on the lag load (the first term, which is a function of *d_ij_*) and the standing genetic load (the second term, which is a function of *V_ij_*). We see that the first term tends to dominate when the population is relatively far from its environmental optimum ($2d_{ij}^{2}\gg V_{ij}$), in which case we have:

$$\mathrm{var}\left[ \ln\left( w_{ij} \right) \right]\approx\frac{d_{ij}^{2}V_{ij}}{\omega_{i}^{2}}$$

As the population approaches the optimum (*d_ij_* 🡪 0), the second term comes to dominate and we have:

$$\mathrm{var}\left[ \ln\left( w_{ij} \right) \right]\approx\frac{V_{ij}^{2}}{2\omega_{i}^{2}}$$

**Genetic covariances/correlations between environments**

The genetic covariance for fitness between two different environmental treatments is given by:

$$\mathrm{cov}\left[ \ln\left( w_{ij} \right),\ln\left( w_{kl} \right) \right]=\mathrm{cov}\left[ \frac{\left( \theta_{i}-z_{ij} \right)^{2}}{2\omega_{i}},\frac{\left( \theta_{k}-z_{kl} \right)^{2}}{2\omega_{k}} \right]=\frac{\mathrm{cov}\left[ d_{ij}\left( z_{ij}-\bar{z}_{ij} \right)-\frac{\left( z_{ij}-\bar{z}_{ij} \right)^{2}}{2},d_{kl}\left( z_{kl}-\bar{z}_{kl} \right)-\frac{\left( z_{kl}-\bar{z}_{kl} \right)^{2}}{2} \right]}{\omega_{i}\omega_{k}}=\frac{\mathrm{cov}\left( z_{ij},z_{kl} \right)\left( 2d_{ij}d_{kl}+\mathrm{cov}\left( z_{ij},z_{kl} \right) \right)}{2\omega_{i}\omega_{k}}$$

where $d_{ij}=\theta_{i}-\bar{z}_{ij}$ and $d_{kl}=\theta_{kl}-\bar{z}_{kl}$; the final result makes use of the identities cov(*X*^2^, *Y*) = 0 and cov(*X*^2^, *Y*^2^) = 2[cov(*X*, *Y*)]^2^, in which *X* and *Y* are standard normal random variables (see Joarder 2009).

When both populations are sufficiently maladapted so that $2d_{ij}d_{kl}\gg\mathrm{cov}\left( z_{ij},z_{kl} \right)$, the covariance is approximately:

$$\mathrm{cov}\left[ \ln\left( w_{ij} \right),\ln\left( w_{kl} \right) \right]\approx\frac{\mathrm{cov}\left[ d_{ij}\left( z_{ij}-\bar{z}_{ij} \right),d_{kl}\left( z_{kl}-\bar{z}_{kl} \right) \right]}{\omega_{i}\omega_{k}}=\frac{d_{ij}d_{kl}\mathrm{cov}\left( z_{ij},z_{kl} \right)}{\omega_{i}\omega_{k}}$$

When one or both populations are near optimum, so that $2d_{ij}d_{kl}\approx0$, we have:

$$\mathrm{cov}\left[ \ln\left( w_{ij} \right),\ln\left( w_{kl} \right) \right]\approx\frac{\left[ \mathrm{cov}\left( z_{ij},z_{kl} \right) \right]^{2}}{2\omega_{i}\omega_{k}}$$

The genetic correlation between environments is:

$$\rho_{ij,kl}=\frac{\mathrm{cov}\left[ \ln\left( w_{ij} \right),\ln\left( w_{kl} \right) \right]}{\sqrt{\mathrm{var}\left[ \ln\left( w_{ij} \right) \right]\mathrm{var}\left[ \ln\left( w_{kl} \right) \right]}}$$

***Three types of genetic covariance/correlation between environments:***

1. For genetic covariances between two different developmental environments (*i* = 1, 2; *j* is shared), $\mathrm{cov}\left( z_{ij},z_{kl} \right)=\mathrm{cov}\left( z_{1j},z_{2j} \right)=V_{x}+b_{j}^{2}V_{g}$

When one or both populations are near optimum, then…

$$\mathrm{cov}\left[ \ln\left( w_{1j} \right),\ln\left( w_{2j} \right) \right]\approx\frac{\left( V_{x}+b_{j}^{2}V_{g} \right)^{2}}{2\omega_{1}\omega_{2}}$$

$$\rho_{1j,2j}\approx\frac{\left( V_{x}+b_{j}^{2}V_{g} \right)^{2}}{V_{1j}V_{2j}}$$

When both populations are maladapted, then…

$$\mathrm{cov}\left[ \ln\left( w_{1j} \right),\ln\left( w_{2j} \right) \right]\approx\frac{d_{1j}d_{2j}\left( V_{x}+b_{j}^{2}V_{g} \right)}{\omega_{1}\omega_{2}}$$

$$\rho_{1j,2j}\approx\frac{d_{1j}d_{2j}\left( V_{x}+b_{j}^{2}V_{g} \right)}{\left| d_{1j}d_{2j} \right|\sqrt{V_{1j}V_{2j}}}$$

1. For genetic covariances between two different parental environments (*j* = 1, 2; *i* is shared), $\mathrm{cov}\left( z_{ij},z_{kl} \right)=cov\left( z_{i1},z_{i2} \right)=V_{x}+V_{yi}+b_{1}b_{2}V_{g}$

When one or both populations are near optimum, then…

$$\mathrm{cov}\left[ \ln\left( w_{i1} \right),\ln\left( w_{i2} \right) \right]\approx\frac{\left( V_{x}+V_{yi}+b_{1}b_{2}V_{g} \right)^{2}}{2\omega_{i}^{2}}$$

$$\rho_{i1,i2}\approx\frac{\left( V_{x}+V_{yi}+b_{1}b_{2}V_{g} \right)^{2}}{V_{i1}V_{i2}}$$

When both populations are maladapted, then…

$$\mathrm{cov}\left[ \ln\left( w_{i1} \right),\ln\left( w_{i2} \right) \right]\approx\frac{d_{i1}d_{i2}\left( V_{x}+V_{yi}+b_{1}b_{2}V_{g} \right)}{\omega_{i}^{2}}$$

$$\rho_{i1,i2}\approx\frac{d_{i1}d_{i2}\left( V_{x}+V_{yi}+b_{1}b_{2}V_{g} \right)}{\left| d_{i1}d_{i2} \right|\sqrt{V_{i1}V_{i2}}}$$

1. or genetic covariances between different developmental and parental environments (*i* = 1, 2; *j* = 1, 2), $\mathrm{cov}\left( z_{ij},z_{kl} \right)=V_{x}+b_{j}b_{l}V_{g}$

When one or both populations are near optimum, then…

$$\mathrm{cov}\left[ \ln\left( w_{11} \right),\ln\left( w_{22} \right) \right]\approx\mathrm{cov}\left[ \ln\left( w_{12} \right),\ln\left( w_{21} \right) \right]\approx\frac{\left( V_{x}+b_{1}b_{2}V_{g} \right)^{2}}{2\omega_{1}\omega_{2}}$$

$$\rho_{11,22}=\frac{\left( V_{x}+b_{1}b_{2}V_{g} \right)^{2}}{V_{11}V_{22}}$$

$$\rho_{12,21}=\frac{\left( V_{x}+b_{1}b_{2}V_{g} \right)^{2}}{V_{12}V_{21}}$$

When both populations are maladapted, then…

$$\mathrm{cov}\left[ \ln\left( w_{11} \right),\ln\left( w_{22} \right) \right]\approx\frac{d_{11}d_{22}\left( V_{x}+b_{1}b_{2}V_{g} \right)}{\omega_{1}\omega_{2}}$$

$$\mathrm{cov}\left[ \ln\left( w_{12} \right),\ln\left( w_{21} \right) \right]\approx\frac{d_{12}d_{21}\left( V_{x}+b_{1}b_{2}V_{g} \right)}{\omega_{1}\omega_{2}}$$

$$\rho_{11,22}=\frac{d_{11}d_{22}\left( V_{x}+b_{1}b_{2}V_{g} \right)}{\left| d_{11}d_{22} \right|\sqrt{V_{11}V_{22}}}$$

$$\rho_{12,21}=\frac{d_{12}d_{21}\left( V_{x}+b_{1}b_{2}V_{g} \right)}{\left| d_{12}d_{21} \right|\sqrt{V_{12}V_{21}}}$$

**References**

Barton NH, Turelli M. (2004). Effects of genetic drift on variance components under a general model of epistasis. *Evolution* 58:2111-2132.

Connallon T, Matthews G.M. (2019). Cross‐sex genetic correlations for fitness and fitness components: Connecting theoretical predictions to empirical patterns. *Evol. Lett.* 3:254-262.

Joarder AH. 2009. Moments of the product and ratio of two correlated chi-square random variables. *Stat. Pap.* 50:581-592.

Tachida H, Cockerham CC. 1988. Variance components of fitness under stabilizing selection. *Genetical Research* 51:47-53.

Wright S. 1935. The analysis of variance and the correlations between relatives with respect to deviations from an optimum. *J Genet.* 30:243-256.

**Supplementary tables and figures**

**Table S1.** Maternal environmental effects on offspring survival at current (16.5°C) and projected (24°C) fertilisation and developmental temperatures. Effects of fertilisation at 16.5°C are shown in blue, and effects of fertilisation at 24°C are shown in pink. Developmental temperatures are rows and columns within each colour. Estimates are ± 1 standard error, with significant estimates in bold.

| *Maternal environmental variance and covariance* | | | | | |
| --- | --- | --- | --- | --- | --- |
|  | *16.5°C* | *24°C* |  | *16.5°C* | *24°C* |
| *16.5°C* | 0.000 ± 0.001 |  | *16.5°C* | 0.001 ± 0.001 |  |
| *24°C* | 0.000 ± 0.001 | 0.000 ± 0.001 | *24°C* | 0.001 ± 0.001 | **0.005** ± **0.002** |


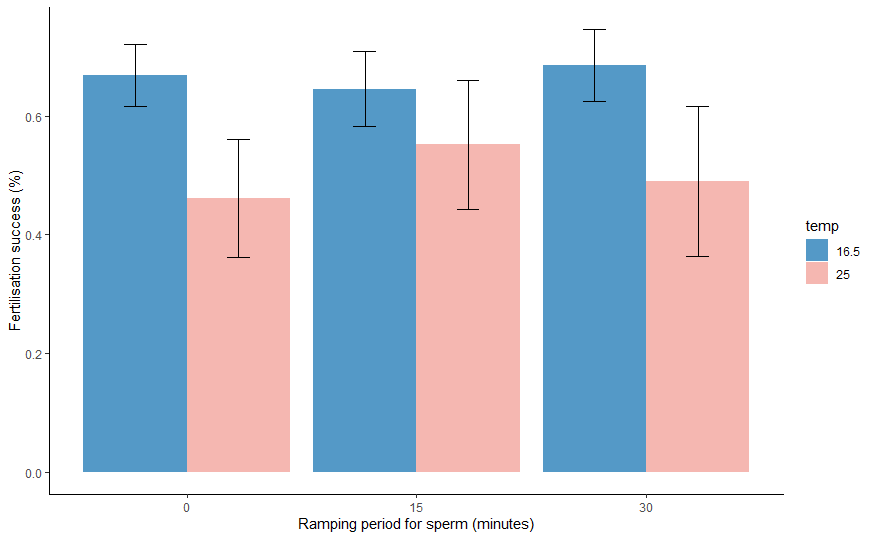


**Figure S1**. No impact of sperm ramping period on mean fertilisation success (±S.E.). Vials of sperm at concentrations of 10^7^ cells ml^-1^ were ramped to treatment temperatures (16.5°C in blue or 25°C in pink) over 0, 15, or 30 minutes. Ramping period did not affect fertilisation success (χ^2^ = 0.17, d.f. = 2, *P* = 0.92).
